# Subtotal colectomy with ileorectal anastomosis (SCIRA) in pediatric intestinal pseudo-obstruction (PIPO): a viable option for patients with frequent enterostomy-related complications?

**DOI:** 10.1007/s00383-025-06010-0

**Published:** 2025-04-18

**Authors:** Anne E. Petersen, Merit Tabbers, Marc A. Benninga, Joep P. M. Derikx, Ramon R. Gorter, Justin R. de Jong

**Affiliations:** 1 Department of Pediatric Surgery, Emma Children’s Hospital, Amsterdam University Medical Centers, University of Amsterdam, Location AMC, Mailbox H7-270, Meibergdreef 9, 1105 AZ Amsterdam, The Netherlands; 2 Amsterdam Gastroenterology Endocrinology Metabolism Research Institute, Amsterdam, The Netherlands; 3 Tytgat Institute for Liver and Intestinal Research, Amsterdam UMC, University of Amsterdam, Amsterdam, The Netherlands; 4 Amsterdam Reproduction and Development Research Institute, Amsterdam, The Netherlands; 5 Department of Pediatric Gastroenterology and Nutrition, Emma Children’s Hospital, Amsterdam University Medical Centers, University of Amsterdam, Location AMC, Amsterdam, The Netherlands

**Keywords:** Pediatric, Pseudo-obstruction, Enterostomy, Subtotal colectomy, Outcomes

## Abstract

**Purpose:**

This study aimed to evaluate the clinical outcomes of subtotal colectomy with ileorectal anastomosis (SCIRA) as an alternative treatment strategy for patients with pediatric intestinal pseudo-obstruction (PIPO) who experienced frequent enterostomy-related complications.

**Methods:**

This retrospective observational study included PIPO patients who underwent SCIRA at our tertiary referral center between 2018 and 2023. Main outcomes were postoperative complications, surgical reinterventions, and the need for enterostomy replacement.

**Results:**

Five patients underwent SCIRA, including four males and one female, at a median age of 14 years (range 6–20). Before SCIRA, all patients had an enterostomy and a history of multiple enterostomy-related complications, requiring a median of nine surgical reinterventions (range 2–13). After SCIRA, the median number of complications was 1 (range 0–2), and the median number of surgical reinterventions was 1 (range 0–2). None of the patients required replacement of their enterostomy. Median follow-up duration was 49 months (range 32–58).

**Conclusion:**

Following SCIRA, a low number of complications and reinterventions was observed, indicating that SCIRA may be a viable option for PIPO patients with frequent enterostomy-related complications. This approach may help to reduce the need for multiple surgical procedures in this challenging patient population.

## Introduction

Pediatric intestinal pseudo-obstruction (PIPO) is a rare and severe form of gastrointestinal dysmotility, in which the structure and/or function of the intestinal neuromusculature is impaired [1]. Due to the neuromuscular impairment, the gastrointestinal tract is unable to propel its contents. This results in clinical features of a mechanical bowel obstruction, including bilious vomiting, failure to pass gas and stool and progressive abdominal distension, yet without any organic occlusion of the lumen [2]. The incidence in children is estimated to range between one per 40,000 and 100,000 live births [3]. Around 5–6% of PIPO patients die during childhood, while the remainder endure significant morbidity and frequent hospitalizations throughout their lives [3, 4].

The management of PIPO is complex and challenging. Advances in parenteral nutrition and bowel decompression techniques have contributed to improved survival rates [3]. The European Society of Pediatric Gastroenterology Hepatology and Nutrition (ESPHGAN) expert group strongly recommends a multidisciplinary expertise team to address each single aspect of the disease. Treatment primarily involves optimizing nutrition, pharmacological therapy and surgery. About 80% of PIPO patients require partial or total parenteral nutrition. Therefore, surgery for central venous catheter (CVC) (re)placement can be necessary [4, 5]. In addition, the ESPHGAN expert group recommends considering the formation of a venting/feeding gastrostomy or jejunostomy and a decompressive ileostomy for all PIPO patients on parenteral nutrition. However, they note that the number of surgical interventions in PIPO patients should be minimized in order to avoid potential complications [3]. This contrasts with the fact that although enterostomy seems to provide the greatest symptom relief, it has been associated with a high complication rate in children with gastrointestinal motility disorders, often resulting in frequent surgical reinterventions [6].

Recently, at our (inter)national expertise center for PIPO, an alternative surgical procedure has been introduced for children with PIPO who experienced frequent enterostomy-related complications. In these patients, subtotal colectomy with ileorectal anastomosis (SCIRA), with or without formation of an antegrade continence enema (ACE), was considered after discussion within the multidisciplinary team, as well as comprehensive consultation of the patients and their parents. In this article, we review our experience with this alternative approach in five PIPO patients, evaluating the clinical outcome in terms of surgical complications and surgical reinterventions.

## Methods

### Study design and population

This is a retrospective study including patients who were diagnosed with PIPO and underwent SCIRA between January 2018—when the procedure was first introduced—and November 2023. This study was performed in the pediatric abdominal tertiary referral center of the Emma Children’s Hospital Amsterdam UMC in the Netherlands, a center of expertise for rare and complex intestinal motility disorders. All patients were diagnosed with PIPO by a pediatric gastroenterologist and/or surgeon, after excluding other organic diseases. Diagnosis was based on the fulfillment of at least two of the four diagnostic criteria established by the ESPHGAN [3]: (I) objective measure of small intestinal neuromuscular involvement; (II) recurrent and/or persistently dilated loops of small intestine with air fluid levels; (III) genetic and/or metabolic abnormalities definitively associated with PIPO; (IV) inability to maintain adequate nutrition and/or growth on oral feeding. For each patient, the multidisciplinary team considered SCIRA due to frequent enterostomy-related complications. The decision regarding ACE formation was based on the need for additional bowel decompression or the expected necessity to perform mechanical irrigation for symptom relief.

### Surgical technique

The surgical technique used consists of (1) SCIRA, with or without (2) ACE formation, and was performed by one or two pediatric surgeons with expertise in gastrointestinal motility disorders.

(1) Preoperative antibiotic prophylaxis according to local protocol was administered. Either a hand-assisted laparoscopic or open approach was used, depending on the history of abdominal surgery of the patient. The colon was fully mobilized (close colon technique) and resected (and send to the histopathological department). Ileorectal anastomosis was performed either hand sewn or using stapling devices. Postoperatively a transanal drain was placed and left in situ for 3 to 5 days postoperatively to ensure adequate passage of gas/stool from the abdomen. Postoperative antibiotics were continued for 48 h if ACE formation was performed.

(2) ACE formation consists of initial placement of a pigtail catheter and replacement for Chait© button after 6 weeks [7]. For this, the last part of the ileum (± 10–20 cm proximal from the ileorectal anastomosis) was fixated to the abdominal wall using three to four stitches. Centrally, a pigtail catheter was placed. Correct placement was checked perioperatively. Antegrade irrigation was initiated on the third day postoperatively.

### Data collection

Data were extracted from the medical records by one researcher (AEP) and then verified by one of the senior authors. Data of interest included (1) demographic and clinical characteristics (i.e., gender, age (years), presence of genetic mutation, accompany of enlarged bladder), (2) clinical course before the procedure (enterostomy creation, complications, and parenteral nutrition), (3) details on the procedure of SCIRA (i.e., surgical approach, duration of hospitalization (days), ACE formation, and complications related to ACE) and (4) clinical course after SCIRA (i.e., complications, defecation frequency (times/day), and follow-up duration (months)).

### Outcomes

The main outcomes of this study were the median number of postoperative complications and surgical reinterventions related to SCIRA and ACE formation per patient, as well as the number of patients requiring enterostomy replacement. Complications were defined as any deviation from the planned clinical course. Severity of complications was assessed using the Clavien–Madadi grading system, a tool recently adapted for pediatric patients that, similar to the Clavien–Dindo grading system, objectively ranks complications occurring within 30 days post-surgery based on the required intervention [8–10]. Complications within 30 days postoperatively were categorized as early and complications beyond 30 days postoperatively as late. In the absence of a standard classification for complications occurring beyond 30 days postoperatively, we applied the Clavien–Madadi scale to both early and late complications. Other outcomes included iatrogenic injury, duration of hospitalization, way of feeding, and defecation frequency.

### Analysis

Due to the nature of this study, only descriptive statistics are presented. Outcomes are reported for each individual patient, as well as the total N, along with the median and range.

### Ethics

The Medical Ethical Committee of the Amsterdam University Medical Centers, Amsterdam, The Netherlands approved the study protocol. In accordance with current regulations, parents of patients under 16 years of age, as well as patients aged 12–16 years, gave written informed consent to the processing of personal data.

## Results

### Patient characteristics (Table 1)

**Table 1:** Patient characteristics and clinical course before SCIRA

	Patient #1	Patient #2	Patient #3	Patient #4	Patient #5
Age (y)	14	11	8	6	20
Sex	Male	Male	Male	Female	Male
PIPO-related genetic mutation	None	None	ACTG2-mutation	ACTG2-mutation	Not tested at patient’s request
Enlarged bladder	No	Yes	Yes	Yes	Yes
Ileostomy; age at procedure (y)	Yes; 11	Yes; 0	Yes; 5	Yes; 2	Yes; 13
Other enterostomy; age at procedure (y)	Jejunostomy; 7	No	No	Gastrostomy; 2	No
Complications associated with enterostomy	High-output stoma	Granulation tissue^a^, intermittent obstruction, retraction^a^	Prolapse, angulation, torsion	High-output stoma, prolapse	Retraction^a^, torsion, prolapse, intermittent obstruction
Surgical interventions (n)	2	9	8	11	13
Parenteral nutrition	0%	0%	100%	100%	100%

^a^Leading to stomal leakage

Five patients were included in this study: four males and one female, with a median age of 14 years (range 6–20) at the time of SCIRA. Baseline characteristics and their clinical course prior to SCIRA details are shown in Table 1. Two patients had a genetically confirmed diagnosis of megacystis microcolon intestinal hypoperistalsis syndrome (MMIHS), by the presence of the enteric smooth muscle actin gamma 2 (ACTG2) mutation. Two other patients met the clinical criteria for MMIHS, although genetic testing either did not identify an underlying PIPO-related genetic abnormality or was not performed at the patient’s request. The fifth patient did not have an enlarged bladder, and no PIPO-related genetic abnormalities were detected, but met the diagnostic criteria for PIPO.

### Clinical course before SCIRA (Table 1)

Prior to undergoing SCIRA, all patients had an ileostomy and required frequent surgical interventions due to enterostomy-related complications, with a median of nine interventions (range 2–13). Indications for these interventions included fascial-level stenosis, torsion, retraction, prolapse, and enterostomy care related problems. Four out of five patients needed parenteral nutrition (PN) in their lives prior to SCIRA, with three patients still dependent on PN at the time of the procedure.

### Main outcomes (Table 2)

**Table 2:** Peri- and post-operative outcomes of SCIRA

	Patient #1	Patient #2	Patient #3	Patient #4	Patient #5
Surgical approach	Hand-assisted laparoscopic	Hand-assisted laparoscopic	Hand-assisted laparoscopic	Median laparotomy	Hand-assisted laparoscopic
ACE formation	No	No	Yes	Yes	No
Iatrogenic injury	Yes, intestinal	No	No	No	Yes, bladder
Hospitalization (d)	5	7	7	9	11
Total no. complications (n)	1	1	2	0	2
Early complications (CM-classification)	Anastomotic stenosis (3B^a^)	None	None	None	Hematoma (1B^b^)
Late complications (CM-classification)	None	Incisional hernia (3B^c^)	Internal herniation (3A^d^); angulation (3B^e^)	None	Incisional hernia (1A^f^)
Surgical reinterventions (n)	1	1	2	0	0
Time to reintervention (m)	1	47	32; 36	N/A	N/A
Parenteral nutrition	0%	0%	100%	100%	100%
Defecation frequency (n/d)	6	3	6	5	7
Follow-up duration (m)	58	56	49	34	32

*CM* Clavien–Madadi, *N/A* not applicable, *d* days, *m* months, *n* number

^a^Revision of anastomosis through laparotomy

^b^Relieved under local anesthesia

^c^Correction through laparotomy

^d^Laparoscopic closure of mesenteric defect

^e^Fixation of the distal ileum to the abdominal wall through laparotomy

^f^Conservatively managed

Four out of five patients (80%) experienced a median of 1 complication (range 0–2), with a median of 1 surgical reintervention (0–2). Complications requiring surgical reintervention included anastomotic stenosis, internal herniation, small bowel stricture and incisional hernia. Early complications (*n* = 2) included subcutaneous hematoma, which was relieved under local anesthesia (CM 1B); and anastomotic stenosis, which was treated by surgical revision of the anastomosis through laparotomy (CM 3B). Late complications (*n* = 4) included internal herniation, treated laparoscopically by closing the mesenteric defect (CM 3A); small bowel angulation, managed by surgical fixation of the distal ileum to the abdominal wall through laparotomy (CM 3B); and two cases of incisional hernia, with one patient opting for surgical repair for cosmetic reasons (CM 3B), while the other choose not to perform surgery (CM 1A). None of the patients required replacement of their enterostomy.

### Other outcomes (Table 2)

Median hospitalization after the procedure was 7 days (range 5–11). In all the patients, the way of feeding before and after SCIRA remained the same: two patients had normal oral intake and three patients were fully dependent on PN. Median defecation frequency after SCIRA was six times per day (range 3–7). Median follow-up duration was 49 months (range 32–58).

### Details on procedure (Table 2)

All patients underwent SCIRA as described in “Methods”. Two patients experienced iatrogenic injuries during SCIRA, both treated during the same procedure. One patient with a stomal loop fixation sustained an intestinal lesion when the abdominal fascia was dissected, which was repaired with dissolvable sutures. Another patient, with prior bladder reduction surgery—resulting in the bladder being adhered to the abdominal wall—sustained a bladder lesion during abdominal entry via Pfannenstiel incision, which was repaired similarly.

### ACE formation

ACE formation was performed in two patients: as a subsequent procedure in patient #3 and simultaneously with SCIRA in patient #4. After undergoing SCIRA, in patient #3, rectal irrigation and gastric drainage were required to address episodes of nausea, biliary vomiting, and abdominal distension. To improve long-term symptom control, the multidisciplinary team decided to perform ACE formation along with gastrostomy placement, which was performed 1 year after SCIRA. Based on this experience, ACE formation was performed simultaneously with SCIRA in patient #4, in whom an outlet problem was expected. In both patients, ACE is used daily and effectively facilitates fecal evacuation. No ACE-related complications were observed during follow-up.

## Discussion

This study reports on the number of postoperative complications and need for surgical reinterventions following SCIRA, an alternative surgical strategy for PIPO patients facing frequent enterostomy-related complications. Over a follow-up period of more than 2 years, a relatively low number of complications and reinterventions were observed and none required stoma reversal. These findings suggest that SCIRA may be a viable option for PIPO patients with frequent enterostomy-related complications, potentially reducing the need for multiple surgery.

Our objective of performing SCIRA was to provide an alternative approach for children with PIPO who experience multiple enterostomy-related complications. A high rate of enterostomy-associated complications was observed prior to SCIRA, which aligns with existing literature indicating that enterostomies in children are generally associated with significant morbidity [11]. Moreover, in a previous study, we found that children with motility disorders are particularly prone to more frequent and more severe enterostomy-related complications compared to children without motility disorders [6]. Nevertheless, the ESPHGAN expert group recommends considering the formation of a venting/feeding gastrostomy or jejunostomy and decompressive ileostomy for all PIPO patients on parenteral nutrition [3]. While enterostomy may provide significant symptom relief, its associated morbidity underscores the need for an alternative approach for these children. For this reason, SCIRA was introduced at our center for children facing frequent enterostomy-related complications. It is important to note that SCIRA was not implemented with the aim of reducing PIPO symptoms but rather as a means to address these complications. Other surgical options mentioned in the ESPHGAN guideline are intestinal transplantation, which should be considered when PN is no longer tolerated. Due to its major risks, we do not consider intestinal transplantation as a feasible alternative of SCIRA in case of enterostomy-related complications.

To date, SCIRA has not been previously described as surgical option for PIPO patients, limiting direct comparisons with existing data for this population. However, outcomes of this technique have been documented in both adults and children with severe slow-transit chronic idiopathic constipation (STIC), for whom subtotal colectomy is considered a surgical treatment of last resort [12]. A systematic review of surgical options for adults with STIC, predominantly involving SCIRA, reported a postoperative complication rate of 24% among 2045 patients, with 13% requiring surgical reintervention and 0.4% experiencing procedure-related mortality [13]. Similarly, a small pediatric study on colonic resection for STIC reported peritonitis in two out of six patients (33%) following SCIRA, both requiring surgical reinterventions [14]. In addition, two patients (33%) in that study required permanent stomas. In contrast, none of the patients in our study required stoma reversal. Although PIPO and STIC have distinct pathophysiological mechanisms, the observed complication rates and surgical reintervention rates in our study align with those reported for STIC. This suggests that, despite the small sample size, the number of complications is representative, supporting its potential role as a viable treatment option for PIPO patients experiencing multiple enterostomy-related complications.

The rarity and complexity of PIPO, along with the heterogeneity among affected patients, highlight the importance of individualized treatment strategies developed by a multidisciplinary team within specialized PIPO expertise centers. SCIRA is tailor-made care and could be considered for patients with PIPO who experience a second or subsequent enterostomy-related complication, including retraction, prolapse, torsion, or stricture, which significantly impact the patient’s quality of life or require surgical intervention. Eligibility for SCIRA should be evaluated within a multidisciplinary team, ensuring a comprehensive evaluation of the patient’s overall condition, surgical history, and anticipated outcomes. In addition, the decision to proceed with SCIRA should involve a shared decision-making process with patient and parents, with discussion of the potential benefits and risks of SCIRA. This approach facilitates informed decision-making and ensures that SCIRA is tailored to the patient’s specific clinical needs.

The primary advantage of SCIRA is the elimination of enterostomy-related complications, thereby reducing the need for additional surgical interventions. In addition, SCIRA restores digestive tract continuity, which is generally associated with improved quality of life. While enterostomies are sometimes necessary and can provide symptom relief, they also present significant challenges, including body image concerns, difficulties in sexual relationships, reduced social functioning, stigmatization, and lowered self-esteem, all of which can contribute to emotional distress [15]. In adults, enterostomies have been associated with substantial deterioration in body image [16], a concern that may be even more pronounced in children and adolescents approaching puberty, when physical appearance and self-perception become increasingly important. Although specific data on PIPO patients are lacking, it is reasonable to assume that these challenges similarly affect this population.

A potential drawback of SCIRA is an increase in defecation frequency, with patients often defecating more than five times per day. This may elevate the risk of involuntary stool loss. Notably, adhesions from multiple prior surgeries in PIPO patients can increase procedural complexity and risks, as reflected by the iatrogenic injuries observed in our study (occurring in two out of five patients). Indeed and recommended by the ESPGHAN expert group, minimizing the number of surgical interventions in PIPO patients is critical [3]. Therefore, SCIRA may warrant consideration early in the treatment pathway of PIPO patients, if enterostomy-related complications occur. Further research is needed to evaluate whether SCIRA could serve as primary treatment for PIPO patients.

Following SCIRA, some patients may experience impaired fecal evacuation, necessitating ACE formation. Importantly, ACE was well tolerated in our cohort, with no procedure-related complications observed during follow-up. Several types of ACE have been steadily used in children with anorectal malformations, spinal dysraphism, and STIC [17], demonstrating improvements in health-related quality of life and high satisfaction rates regardless of ACE technique [18]. The decision to perform ACE placement as part of SCIRA has been an evolving aspect of our clinical approach. To minimize the need for multiple abdominal surgeries, ACE formation should ideally be performed during the primary procedure. However, the precise indications for ACE remain individualized and require a multidisciplinary evaluation. The option of ACE should be discussed as part of the treatment plan, considering rectal irrigation as a possible alternative. In cases where ACE is not initially placed, clinicians should anticipate that rectal irrigation may be required for bowel management. Conversely, if an ACE is placed but later deemed unnecessary, it can be reversed. The advantages and disadvantages of each approach should be carefully weighed in shared decision-making with the patient and their caregivers.

This study’s strengths include the use of definitions from the ESPGHAN guidelines, the classification of complications according to Clavien–Madadi and follow-up duration of at least 3 years for each patient. However, our study has limitations, including the small group of patients and the lack of information on patient satisfaction and overall health-related quality of life, which are essential for evaluating the overall outcome of this alternative treatment strategy. In addition, extended follow-up periods are needed to assess the long-term outcomes.

## Conclusion

Given the low number of complications and surgical reinterventions, SCIRA could be a viable alternative for PIPO patients who have experienced frequent enterostomy-related complications. Further studies should focus on evaluating patient satisfaction and long-term outcomes.

## Data Availability

No datasets were generated or analyzed during the current study.
